# A Doctor’s Life

**Published:** 1856-02

**Authors:** 


*A Doctor's Life. By an M.D., of Alna Mich.*

The following are some of the sweets of a Dr.'s life. If he visits a few of his customers when they are well, it is to get his dinner; if he don't do so, it is because he cares more about the fleece than the flock. If he goes to church regularly, it is because he has nothing else to do; if he don't go, it is because he has no respect for the Sabbath or religion. If he speaks to a poor person, he keeps bad company; if he passes them by, he is a better man than other folks. If he has a good carriage, he is extravagant; if he uses a poor one, on the score of economy, he is deficient in necessary pride. If he makes parties, it is to soft-soap the people to get their money; if he don't make them, he is afraid of a *cent!* If his horse is fat, it is because he has nothing to do; if he is lean, it is because he isn't taken care of. If he drives fast, it is to make the people think somebody is very sick; if he drives slow, he has no interest in the welfare of his patients. If he dresses neat, he is proud; if he does not, he is wanting in self-respect. If he works on the land, he is fit for nothing but a farmer; if he don't work, it is because he is too lazy to be anything. If he talks much, " we don't want a Dr. to tell everything he knows;" if he don't talk, "*we like to see a Dr. social.*" If he says anything about politics, he had better let it alone; if he don't say anything about it, " we like to see a man show his colors." If he visits his patients every day, it is to run up a bill; if he don't, it is unjustifiable negligence. If he says anything about religion, he is a hypocrite; if he don't, he is an infidel. If he uses any of the popular remedies of the day, it is to cater to the whims and prejudices of the people to fill his pockets; if he don't use them, it is from professional selfishness. If he is in the habit of having counsel often, it is because he knows nothing; if he objects to having it, on the ground that he understands his own business, he is afraid of exposing his ignorance to his superiors. If he gets pay for one-half his services, he has the reputation of being a *great manager!* Who wouldn't be an M. D.?

*Nashville Jour. of Med. and Surgery*, Oct. 1855.

